# Cost-effectiveness analysis of a quality improvement program to reduce caesarean sections in Brazilian private hospitals: a case study

**DOI:** 10.1186/s12978-021-01147-2

**Published:** 2021-05-08

**Authors:** Rosa Maria Soares Madeira Domingues, Paula Mendes Luz, Barbara Vasques da Silva Ayres, Jacqueline Alves Torres, Maria do Carmo Leal

**Affiliations:** 1 Fundação Oswaldo Cruz, Laboratório de Pesquisa Clínica Em DST/Aids, Instituto Nacional de Infectologia Evandro Chagas, Av. Brasil, 4365, Manguinhos, Rio de Janeiro, RJ CEP 21040-360 Brazil; 2 Fundação Oswaldo Cruz, Instituto Nacional de Infectologia Evandro Chagas, Rio de Janeiro, Brazil; 3 Fundação Oswaldo Cruz, Escola Nacional de Saúde Pública Sérgio Arouca, Rio de Janeiro, Brazil; 4 Agência Nacional de Saúde Suplementar, Rio de Janeiro, Brazil; 5 Fundação Oswaldo Cruz, Escola Nacional de Saúde Pública Sérgio Arouca, Rio de Janeiro, Brazil

**Keywords:** Caesarean delivery, Healthcare quality improvement, Hospitals medicine, Cost-effectiveness

## Abstract

**Background:**

In 2015, a quality improvement project of childbirth care called Adequate Childbirth Project (“Projeto Parto Adequado”- PPA) was implemented in Brazilian public and private hospitals, aiming to improve the quality of childbirth care and to reduce caesarean sections without clinical indications. The objective of this study is to conduct an economic analysis of two models of care existing in a private Brazilian hospital—the model following the recommendations of the PPA and the standard of care model—in reducing the proportion of caesarean sections.

**Methods:**

We conducted a cost-effectiveness analysis using data from one of the private hospitals included in the PPA project. The main outcome was the proportion of caesarean section. We used total cost of hospitalization for women and newborns, from the health care sector perspective, during the length of the observed hospital stay. We did not apply discount rates and inflation rate adjustments due to the short time horizon. We conducted univariate sensitivity analysis using the minimum and maximum costs observed in hospitalizations and variation in the probabilities of caesarean section and of maternal and neonatal complications.

**Results:**

238 puerperal women were included in this analysis. The PPA model of care resulted in a 56.9 percentage point reduction in the caesarean section probability (88.6% vs 31.7%, p < 0.001) with an incremental cost-effectiveness ratio of US$1,237.40 per avoided caesarean section. Women in the PPA model of care also had a higher proportion of spontaneous and induced labor and a lower proportion of early term births. There were no maternal, fetal or neonatal deaths and no significant differences in cases of maternal and neonatal near miss. The cost of uncomplicated vaginal births and caesarean sections was the parameter with the greatest impact on the cost-effectiveness ratio of the PPA model of care.

**Conclusion:**

The PPA model of care was cost-effective in reducing caesarean sections in women assisted in a Brazilian private hospital. Moreover, it reduced the frequency of early term births and did not increase the occurrence of severe negative maternal and neonatal outcomes.

## Plain English summary

Caesarean section is a safe intervention to save the lives of women and newborns. However, it is associated with negative health consequences for women and children in the short, medium and long term. Therefore, its harmful effects can outweigh its benefits when used excessively. Caesarean rates are increasing globally. In Brazil, caesarean section is the most frequent type of birth since 2009.

In 2015, a quality improvement project, called “Adequate Childbirth Project” (PPA), was implemented in Brazilian public and private hospitals with the aim of improving the quality of childbirth care and reducing caesarean sections without clinical indications.

In this study, we compared the caesarean rate and the hospitalization costs of women assisted in this new model of care and in the standard of care model. The women attended in the PPA model of care had a lower rate of caesarean section (31.7% vs 88.6% in the standard of care model), with no differences in severe negative maternal and neonatal outcomes. Women in the PPA model of care also had a higher frequency of spontaneous or induced labor and a lower frequency of early term births, that is, less than 39 weeks of gestation. The PPA model of care was considered cost-effective, that is, it produced good results for the amount of money spent. This is a promising result for services that face the problem of increasing numbers of caesarean sections and are looking for strategies to improve the quality of childbirth care.

## Introduction

Caesarean section (CS) is a safe intervention to save the lives of women and newborns. However, it is associated with negative short, medium and long-term health consequences for women and children [1–5]. Therefore, its harmful effects can outweigh its benefits when used excessively. At the population level, there is no evidence that caesarean rates above 10% are associated with a reduction in the maternal mortality ratio [6], although population characteristics may affect caesarean rates in each country [7]. In Brazil, based on the C-model tool [7], and considering demographic and obstetric characteristics for Brazilian women, the expected rate of caesarean delivery for the country would be 25 to 30% [8]. For women admitted to private hospitals, this value would be 44.4%. The main driver for the expected high CS rate in Brazilian private hospitals is the high frequency of women with a previous CS.

Since 2009, caesarean section is the most frequent type of birth in the country. Caesarean section is associated with demographic, socioeconomic factors and the type of childbirth care services organization. Based on two national surveys [9, 10], the national average of caesarean section rates in women assisted in Brazilian private services reaches 80–90%.

In 2015, the National Supplementary Health Agency (ANS), responsible for regulating the supplementary health sector in the country, together with the Institute for Health Improvement (IHI) and Hospital Israelita Alberto Einstein, and with the support of the Brazilian Ministry of Health, implemented the Adequate Childbirth Project (“Projeto Parto Adequado-PPA”). This is a quality improvement project of childbirth care in Brazilian hospitals that aims to identify innovative and viable models of care, which value vaginal delivery and reduce the percentage of caesarean sections without clinical indication [11, 12].

The objective of this study is an economic analysis of two models of care existing in a private Brazilian hospital—the model following the recommendations of the PPA and the standard of care model—in reducing the proportion of caesarean sections.

## Methods

### Study design

This is an economic analysis of the model of care implemented from the PPA to reduce caesarean sections in a private Brazilian hospital.

**Fig. 1:**
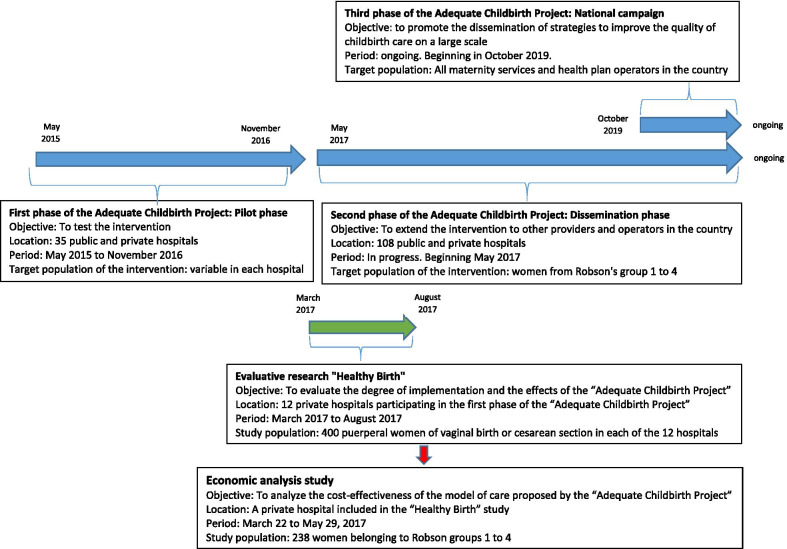
Description of the “Adequate Childbirth Project", the evaluative research "Healthy Birth "and the economic analysis study

The PPA is a quality improvement project of childbirth care implemented in Brazilian public and private hospitals, with the goal of increasing the percentage of vaginal deliveries and reducing the rate of hospitalization in a neonatal intensive care unit (NICU) [11–13]. It is an ongoing project, started in May 2015 and developed in three phases described in Fig. 1. More information about the PPA is available at the ANS website [11] and at Boren et al. [12].

In 2017, an evaluative research called the “Healthy Birth” study was carried out to assess the degree of implementation and the effects of the PPA in a convenience sample of 12 hospitals, among the 23 private hospitals that participated in the first phase of the project [14]. Sample calculation used as parameters a 50% prevalence of cesarean section and a power of 80% to detect a 10% reduction in the caesarean rate with a significance level of 5% resulting in a minimum sample of 400 women in each hospital.

All women admitted for childbirth care and who met the eligibility criteria were invited to participate in the study, until 400 participants were included in each hospital. Face-to-face interviews with puerperal women were conducted at least 6 h after vaginal delivery and 12 h after a caesarean section. Hospital records were reviewed and data were extracted after hospital discharge. Data collection was carried out from March 2017 to August 2017, 6 to 8 months after the end of the first phase of the PPA (Fig. 1). More information about the “Healthy Birth” study is available in Torres et al. [14].

For this economic analysis, we carried out a case study in one of the hospitals included in the “Healthy Birth” study, the only hospital that made available the hospitalization costs of the 400 women included in the study. This hospital is located in the southeastern region of the country and belongs to a private health insurance provider. Figure 1 summarizes the information from the PPA, the “Healthy Birth” study and this economic analysis.

### Comparative models of care: PPA model of care and standard of care model

The PPA project aims to support and promote the implementation of actions to reduce the percentage of unnecessary caesarean sections and increase the quality and safety of care during labor and birth. It is a multifaceted intervention, with activities that are structured in four theoretical components: (1) Governance (coalition between leaders aiming at quality and safety in childbirth care); (2) Women and families (empowerment of women and their families aiming at their active participation in the process of pregnancy, childbirth and postpartum); (3) Reorganization of care (reorganization of care in order to favor the physiological evolution of labor and the performance of caesarean sections based on clinical factors); (4) Monitoring (structuring information systems for continuous learning). The activities provided for in the four components are based on scientific evidence [15] and on 2 successful strategies for reducing caesarean sections in Brazilian private hospitals [16, 17].

The PPA uses the IHI improvement model, where through the cyclical and incremental implementation of changes, the proposed activities are tested and adjusted to the local context, allowing their implementation and refinement [13]. In the first phase of the project, managers and local leaders participated in five face-to-face learning sessions in addition to monthly virtual learning sessions, focusing on training in the improvement model, initial tests of change to reduce the caesarean rate based on the four PPA components and sharing successful experiences and challenges in implementing changes. In addition, the project offered clinical training at realistic simulation centers, with a focus on assisting physiological vaginal delivery and managing obstetric complications [12].

At the hospital level, the project implemented new forms of care organization, with changes in the hospital environment, access to non-pharmacological methods for pain relief and equipment for births in vertical positions. Hospital staff included nurse-midwives in childbirth care and trained professionals implemented clinical guidelines. Activities for women aimed at greater autonomy and involvement of pregnant women in the decision-making process related to childbirth, through access to information, participation in educational groups, encouragement to develop a birth plan and visit to the hospital.

In Brazilian private hospitals, women assisted in the standard of care model are usually seen by the same doctor during prenatal and childbirth care [16]. There is low participation of nurse-midwives [18], a high proportion of antepartum cesarean section [10], low use of labor induction [10], low use of best practices and high use of obstetric interventions during labor [19].

### Study population

Women with live births of any weight or gestational age and stillbirths weighing 500 g or more or with 22 or more gestational weeks were eligible for the “Healthy Birth” study. Exclusions included home births or public deliveries, women with legal determination to terminate pregnancy, and women who did not speak Portuguese or with hearing loss, due to the difficulties in conducting the face-to-face interviews.

For this cost-effectiveness analysis, we restricted the study population to women in the Robson's groups 1 to 4. Robson's classification classifies women into 10 mutually exclusive groups based on the type of pregnancy, parity, fetal presentation, gestational age and in the presence of a previous cesarean section [20]. Groups 1 to 4 correspond to primiparous and multiparous women, with single, full-term pregnancies, with a fetus in cephalic presentation and without previous cesarean section. Groups 2 and 4 can also be subdivided into groups 2a and 2b and 4a and 4b, with groups “a” represented by women with labor induction and groups “b” by women with antepartum caesarean section [21].

Robson’ s groups 5 to 10 have higher probability of caesarean section and we restricted the analysis to groups 1 to 4 to make women assisted in the two models of care more comparable, as the standard of care model had a higher proportion of women in groups 5 to 10. Moreover, women belonging to Robson’ s groups 1 to 4 are the current target population of PPA in all hospitals participating in the project. In this regard, results from this economic analysis would be more applicable to the project’s aim though less applicable in general terms.

We also excluded women with diagnosis of HIV infection and newborns with congenital malformations or with gestational age equal to or greater than 42 weeks, due to the greater possibility of caesarean sections and negative outcomes. As we used the perspective of the health care sector, we also excluded women with out of pocket payment for childbirth care.

In this hospital, all women assisted by the hospital team on duty were followed according to the PPA model of care. Using data obtained during the interview, we classified women as “PPA model of care”, if they reported being assisted by the hospital team on duty, and as “standard of care model”, if they were assisted by an external staff.

### Effectiveness

We used the primary outcome “proportion of caesarean section”, including antepartum and intrapartum caesarean sections, obtained from hospital records, to measure effectiveness. In addition to the crude rate, we used propensity score weighting to obtain a weighted estimate [22], since some maternal characteristics associated with higher rates of caesarean section [10], such as “age”, “years of study”, “initial preference for the type of childbirth” and “pregnancy risk”, showed significant differences between the two models of care.

As secondary outcomes we used maternal outcomes (maternal death, Severe Maternal Morbidity, Maternal Near Miss, admission to an adult Intensive Care Unit, satisfaction with the care received) and perinatal outcomes (birth weight, gestational age at birth, asphyxia in the fifth minute of life, any neonatal complication, NICU admission, neonatal Near Miss, neonatal death and fetal death). For the measurement of Severe Maternal Morbidity (SMM), Maternal Near Miss (MNM) and Neonatal Near Miss (NNM), we used data from hospital records and the case definition criteria recommended by the World Health Organization [23, 24]. To assess satisfaction with hospital care, we used data obtained during the interview and a validated scale for the Brazilian population [25].

### Costs

We used aggregate costs (total cost of hospitalization for women and newborns), from the perspective of the health care sector, within the adopted time horizon, that is, the length of the observed hospital stay. We did not apply discount rates and inflation rate adjustments due to the short time horizon.

In Brazilian private hospitals, there are different arrangements for payment of hospitalizations for childbirth care. The hospital included in this case study used the “package model”. In this model, the health care sector operator defines a basic package that covers the costs related to hospitalizations of vaginal births and caesarean sections without complications, to which are added the costs resulting from complications that may have occurred during hospitalization. The value of the basic package depends on the type of health plan, with a wide variation in the cost of hospitalization of women with vaginal or caesarean delivery without complications. Complication costs are not broken down into the total amount charged by the hospital to the health care sector operator. However, the total cost reported, which we used in this analysis, includes the costs of the vaginal birth or caesarean section and all maternal and neonatal complications that occurred during hospitalization.

We assessed the mean cost and the range of the total cost of hospitalization of vaginal births and caesarean sections, with or without maternal and neonatal complications, for women in each type of model of care: the PPA and the standard of care model. The costs related to the implementation and maintenance of PPA activities were not available.

### Cost-effectiveness analysis

We calculated the incremental cost-effectiveness ratio (ICER) using the difference in the total cost of hospitalization and the difference in the proportion of cesarean sections in the two groups. The cost-effectiveness threshold of 18 to 71% of the Gross Domestic Product (GDP) per capita proposed for middle-high income countries was adopted [26], corresponding to the values of US$1,776.77 to US$7,008.37 for the year 2017 (1US$ = R$3.2).

### Scenario analysis

We conducted univariate sensitivity analysis with the results being presented on a tornado diagram. We used the minimum and maximum costs observed in hospitalizations with and without maternal and neonatal complications in the two models of care. In the group assisted in the PPA model of care, we used variation in the probabilities of maternal and neonatal complications considering the minimum and maximum values observed, and variation in the probability of cesarean section, considering the theoretical plausibility of greater or lesser effects of the PPA model of care. We also used the hypothetical scenario of equal costs of hospitalizations for uncomplicated vaginal births and caesarean sections in both models of care.

We used the statistical package R for analyzes.

## Results

During the study period, 409 women were interviewed. Exclusions included women of Robson’s groups 5 to 10 (164), Robson’ s group not classified (2), newborn with congenital malformation (2), gestational age equal to or greater than 42 weeks (2), women with HIV infection (1), or out-of-pocket payment of the hospitalization (2). Of the 238 included in the analysis, 84 were assisted in the PPA model of care and 154 in the standard of care model.

Women’ s mean age was 29 years old. Most women self-reported as mixed or white, had 11 to 14 years of study, belonged to the higher economic classes [27], lived with a partner and had paid work. Only a fifth of women had a previous delivery, 55.9% preferred a vaginal delivery and 51.3% belonged to Robson's group 2. A fifth of women had a risk pregnancy and 48.3% were overweight or obese. Women assisted in the standard of care model were older, more educated, belonged more to higher economic classes (A and B), had a higher proportion of risk pregnancy and overweight/obesity, and belonged to a greater proportion to Robson's group 2b. Women assisted in the PPA model of care had a greater preference for vaginal delivery and were mostly from Robson's group 1. There were no statistically significant differences in relation to skin color, parity, marital status and paid work (Table 1).

**Table 1:** Demographic, social and obstetric characteristics of women according to the model of care. Rio de Janeiro, 2017

Characteristic	Total (N = 238)		Standard of care model (N = 154)		PPA model of care (N = 84)		p value
	n	%	n	%	n	%	
Age (years)							
< 20	7	2.9	4	2.6	3	3.6	< 0.001
20–34	186	78.2	109	70.8	77	91.7	
35 or more	45	18.9	41	26.6	4	4.8	
Skin color							
White	78	32.8	54	35.1	24	28.6	0.307
Black	45	18.9	25	16.2	20	23.8	
Mixed/Asian/Indigenous	115	48.3	75	48.7	40	47.6	
Schooling (years)							
1 to 10	34	14.3	24	15.6	10	11.9	0.007
11 to 14	136	57.1	76	49.4	60	71.4	
15 or more	52	21.8	40	26.0	12	14.3	
Postgraduate studies	16	6.7	14	9.1	2	2.4	
Economic class							
D/E	1	0.4	0	0.0	1	1.2	0.003
C	70	29.4	36	23.4	34	40.5	
B	156	65.5	107	69.5	49	58.3	
A	11	4.6	11	7.1	0	0.0	
Lives with partner	205	86.1	136	88.3	69	82.1	0.188
Paid work	183	76.9	118	76.6	65	77.4	0.895
Parity							
0	192	80.7	125	81.2	67	79.8	0.957
1 to 2	43	18.1	27	17.5	16	19.0	
3 or more	3	1.3	2	1.3	1	1.2	
Preference for vaginal birth	133	55.9	66	42.9	67	79.8	< 0.001
Robson group							
1	70	29.4	26	16.9	44	52.4	< 0.001
2a	19	8.0	2	1.3	17	20.2	
2b	103	43.3	97	63.0	6	7.1	
3	19	8.0	7	4.5	12	14.3	
4a	5	2.1	3	1.9	2	2.4	
4b	22	9.2	19	12.3	3	3.6	
Risk pregnancy	49	20.6	38	24.7	11	13.1	0.035
Pré-gestational BMI							
Underweight	9	3.8	6	3.9	3	3.6	0.023
Normal	113	47.9	62	40.8	51	60.7	
Overweight	68	28.8	48	31.6	20	23.8	
Obese	46	19.5	36	23.7	10	11.9	

*PPA* adequate birth project, *BMI* Body Mass Index

The proportion of caesarean sections was three times higher in the standard of care model (91.6% vs 32.1%, p < 0.001), with a small difference reduction in the weighted analysis (88.6% vs 31.7%, p < 0.001). There were no maternal deaths. The differences in the proportions of SMM, MNM, admission to an adult ICU and satisfaction with childbirth and newborn care were not significant (Table 2).

**Table 2:** Maternal and neonatal outcomes according to the model of care. Rio de Janeiro, 2017

Maternal and neonatal outcomes	Total (N = 238)		Standard of care model (N = 154)		PPA model of care (N = 84)		p value
	N	%	n	%	n	%	
Maternal outcomes							
Type of labor							
Spontaneous	89	37.4	33	21.4	56	66.7	< 0.001
Induced	24	10.1	5	3.2	19	22.6	
Without labor	125	52.5	116	75.3	9	10.7	
Type of birth							
Vaginal	70	29.4	13	8.4	57	67.9	< 0.001
Caesarean	168	70.6	141	91.6	27	32.1	
Weighted caesarean^a^				88.6		31.7	
Severe maternal morbidity	5	2.1	4	2.6	1	1.2	0.658
Maternal near miss	1	0.4	1	0.7	0	0.0	1.000
Maternal death	0	–	0	–	0	–	
Admission to Intensive Care Unit	5	2.1	5	3.3	0	0.0	0.164
Maternal satisfaction with childbirth care							
Clarity of information	219	92.0	143	92.9	76	90.5	0.517
Respect	227	95.4	147	95.5	80	95.2	0.939
Preserved intimacy	231	97.1	151	98.1	80	95.2	0.220
Time available for questions	212	89.1	141	91.6	71	84.5	0.096
Participation in the decision making	217	91.2	144	93.5	73	86.9	0.086
Any type of violence	6	2.5	3	1.9	3	3.6	0.668
Global satisfaction with childbirth care	228	95.8	148	96.1	80	95.2	0.745
Satisfaction with newborn care	228	95.8	145	94.2	83	98.8	0.103
Neonatal outcomes							
Birth weight							
< 2.500 g	7	3.0	5	3.3	2	2.4	0.225
2500 to 3999 g	218	92.8	144	94.1	74	90.2	
≥ 4000 g	10	4.3	4	2.6	6	7.3	
Gestational age (weeks)							
37 to 38	107	45.0	83	53.9	24	28.6	< 0.001
Spontaneous^b^	35	21.1	17	19.3	18	23.1	0.553
Induced	72	35.5	66	48.2	6	9.1	< 0.001
39 to 41	131	55.0	71	46.1	60	71.4	< 0.001
Apgar < 7 in the 5th min of life	2	0.9	1	0.7	1	1.2	1.000
Any neonatal complication	30	12.6	16	10.4	14	16.7	0.163
Admission to NICU	22	9.2	11	7.1	11	13.1	0.130
Neonatal near miss	19	8.0	11	7.1	8	9.5	0.517
Stillbirth	0	–	0	–	0	–	
Neonatal death	0	–	0	–	0	–	

*PPA* Adequate Birth Project; *NICU* Neonatal Intensive Care Unit

^a^Weighted proportion of cesarean section using the propensity score. The variables “age”, “years of study”, “initial preference for the type of childbirth” and “risk pregnancy” were used as explanatory variables for the logistic model of the propensity score and the type of model of care as the outcome variable

^b^Spontaneous labor or rupture of membranes

There were no fetal or neonatal deaths. Of the total live births, 3% had low birth weight, 55% had gestational age between 39 and 41 weeks, 0.9% had moderate asphyxia in the 5 min of life, 12.6% had some neonatal complication, 9.2% were admitted to the NICU and 8% were classified as NNM. A significantly higher proportion of early term births (gestational age 37–38 weeks) was observed in the standard of care model, with 79.5% of these through obstetric intervention (94.1% in antepartum cesarean sections and 5.6% in induced vaginal deliveries, data not shown in the table). The most frequent neonatal complication in both groups was transient tachypnea. Meconium aspiration syndrome was significantly more frequent in the PPA model of care (3.8% in the PPA care model vs 0 in the standard of care model, p = 0.041). There were no significant differences in the other neonatal outcomes (Table 2).

**Fig. 2:**
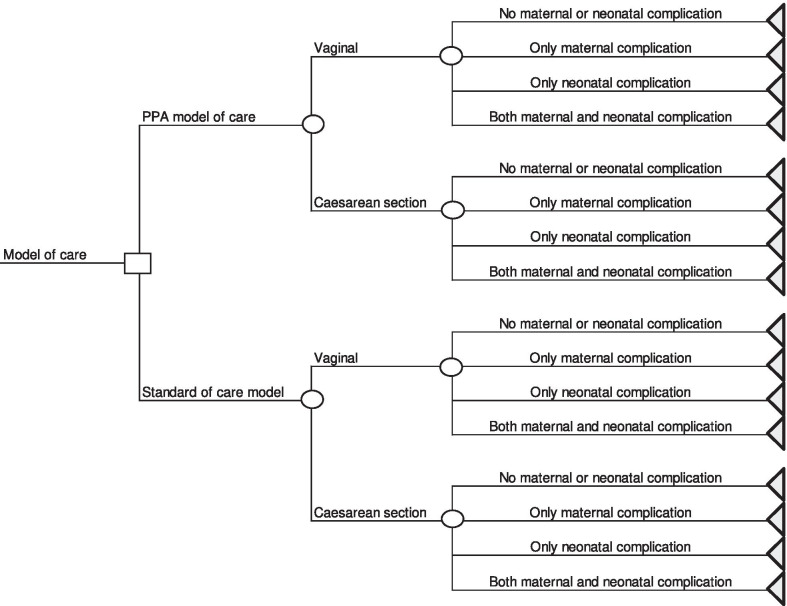
Decision tree diagram used for the cost-effectiveness analysis comparing the two models of care

Women in the PPA model of care were more likely to have maternal complications after a vaginal delivery (0.088) and less likely to have maternal complications after a cesarean section (0.037) than women in the standard of care model (0.077 and 0.050, respectively). The probability of neonatal complications was higher in women in the PPA model of care, both in vaginal delivery (0.105 vs 0.077) and in caesarean section (0.185 vs 0.071). The average cost of hospitalizations without complications was higher in women assisted in the PPA model of care, both for vaginal deliveries (US$1243.22 vs US$1034.20) and for caesarean sections (US$1263.27 vs US$919.78). In hospitalizations with complications, the greatest differences were observed in vaginal births with only neonatal complications, with the average cost in women assisted in the PPA model of care almost twice as high (US$7131.00 vs US$3737.12). Considering the primary outcome of the study, the PPA model of care resulted in a 56.9 percentage point reduction in the caesarean section probability and an increase in the total cost of US$704.10, which implies an incremental cost-effectiveness ratio of US$1237.40 per avoided cesarean section (Table 3 and Fig. 2).

**Table 3:** Probabilities of type of birth, complications and costs of hospitalization according to model of care

Parameters	Standard of care model (n = 154)	PPA model of care (n = 84)
Effectiveness		
Proportion of caesarean^a^	88.6	31.7
Probability of no maternal or neonatal complication after a vaginal birth	0.846	0.825
Probability of only maternal complication after a vaginal birth	0.077	0.070
Probability of only neonatal complication after a vaginal birth	0.077	0,088
Probability of both maternal and neonatal complication after a vaginal birth	0	0.018
Probability of no maternal and neonatal complication after a CS	0.887	0.778
Probability of only maternal complication after a CS	0.043	0.037
Probability of only neonatal complication after a CS	0.064	0.185
Probability of both maternal and neonatal complication after a CS	0.007	0
Costs (mean value)		
Vaginal birth with no maternal or neonatal complication	US$1,034.20	US$1,243.22
Vaginal birth with only maternal complication	US$1,464.38	US$1,243,83
Vaginal birth with only neonatal complication	US$3,737.12	US$7,131,00
Vaginal birth with both maternal and neonatal complication	US$0.0	US$11,280.73
Caesarean with no maternal or neonatal complication	US$918.78	US$1,263.27
Caesarean with only maternal complication	US$2,108.95	US$1,874.69
Caesarean with only neonatal complication	US$5,744.49	US$5.951,62
Caesarean with both maternal or neonatal complication	US$5,068.28	US$0.0
Cost-effectiveness analysis		
Difference in probability of caesarean section		0.569
Difference in total cost		US$704.10

Incremental Cost-Effectiveness Ratio (ICER) US$1,237.40/avoided cesarean section

^a^Weighted proportion of cesarean section using the propensity score. The variables “age”, “years of study”, “initial preference for the type of childbirth” and “risk pregnancy” were used as explanatory variables for the logistic model of the propensity score and the type of model of care as the outcome variable

**Fig. 3:**
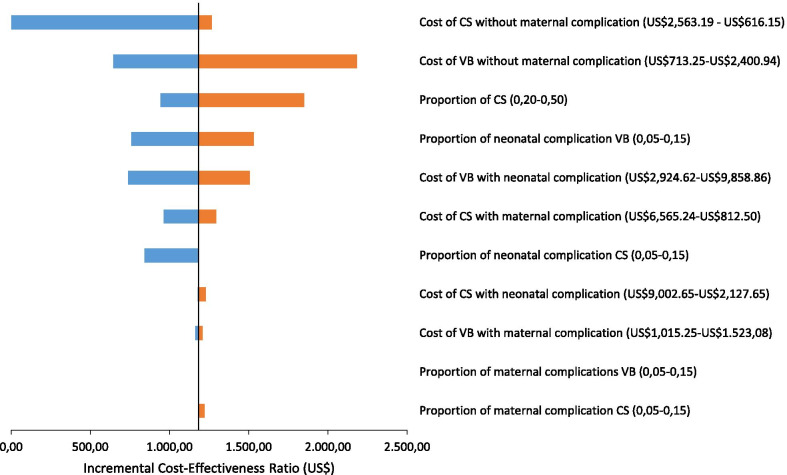
Univariate sensitivity analysis of parameters of cost and complications at the Incremental Cost-Effectiveness Ratio

In the sensitivity analysis, the variation in the cost of caesarean section without maternal complication, applied to both groups, was the parameter with the greatest effect on ICER: when the cost of hospitalization is greater than US$1960.00, women assisted in the PPA model of care have a 56.9 reduction in the proportion of cesarean sections at a lower cost. The increase in the cost of uncomplicated vaginal delivery in both groups and in the proportion of caesarean sections in the PPA group make the PPA model of care less cost-effective, while the reduction in the proportion of neonatal complications in vaginal births in the PPA group and the costs of these hospitalizations in both groups make it more cost-effective. The ICER was less sensitive to variations in the parameters of probability of maternal complications and the cost of these hospitalizations (Fig. 3). The use of the same average cost of hospitalization for vaginal births and caesarean sections without complications in both models of care, with an average cost variation of US$625.00 to US$2,500,00, resulted in a ICER of US$641.20 to US$579.38 per caesarean section avoided (data not shown in table).

## Discussion

The PPA model of care was cost-effective in reducing caesarean sections in a private hospital located in the southeast of Brazil, the most developed region of the country. The Brazilian government does not recommend a threshold value for decisions on the incorporation of new technologies in the National Health System [28]. Therefore, we used a threshold value recommended for the evaluation of interventions in middle-income countries [26]. If we used the threshold of less than 1GDP per effectiveness recommended by the World Health Organization [29], the PPA model of care would be very cost-effective.

The two parameters with the greatest influence on the cost-effectiveness results—the average cost of hospitalizations for vaginal births and caesarean sections without complications—are not related to the model of care. However, they are important parameters in the context of childbirth care in Brazilian private hospitals, since there are different arrangements for paying for hospitalizations in these hospitals. The inpatient package model, used in the hospital included in this study, is the most common. In this model, the health insurance company defines the cost of hospitalizations for uncomplicated vaginal births and caesarean sections with a wide variation of this value according to the type of health plan. In this study, the average cost of uncomplicated vaginal birth and caesarean sections was higher in women assisted in the PPA model of care. Scenario analysis adopting the same average cost of hospitalization for uncomplicated vaginal and caesarean sections in the two models of care, using a wide range of values, resulted in lower ICER values for avoided caesarean section, making the PPA model of care even more cost- effective.

The other parameters found to influence the ICER were the proportion of caesarean sections and the proportion of neonatal complications, especially those related to vaginal deliveries. The greater the reduction in the rate of caesarean section in the PPA model of care, and the lower the occurrence of neonatal complications, the more cost-effective this model of care is. In a scenario of lower reduction in the caesarean section rate, reaching a rate of 50% in the PPA model of care group, the ICER would rise to US$1760.50 per avoided cesarean section. Regarding neonatal complications, the significantly higher proportion of meconium aspiration syndrome in the PPA group indicate that there are possibilities for improving the quality of childbirth care in this model of care.

The 2/3 reduction in the caesarean section rate in the PPA model of care, in both crude and weighted analysis, is consistent with two previous studies that evaluated the effects of interventions to reduce caesarean sections in Brazilian private hospitals, which also found significantly lower rates of caesarean section [16, 17]. All these interventions have as common characteristics the implementation of models of care that promote physiological childbirth through adjustments in the hospital environment, implementation of clinical guidelines based on best clinical practices, promotion of the collaborative model of care between doctor and nurse-midwives during labor and childbirth care, and educational work with women.

The group of women assisted by the standard of care model had a much higher proportion of women in the Robson’s groups 2b and 4b, similar to a previous national study carried out in 2011–2012, where group 2b was the most frequent group in Brazilian private hospitals [30]. In the PPA model of care, groups 1 and 2a were the most frequent. Almost 90% of women in the PPA group presented labor, while in the standard of care model this value was lower than 25%. Finally, in the PPA model of care the proportion of induced labor was seven times higher than that observed in the standard of care model, where the induction rate was less than 5%. All these results suggest that the differences found between the two models of care are due to the actions implemented by the PPA. A more global change in the private sector is less likely, as women assisted by external teams showed the same pattern seen in private hospitals in 2011–2012 [10, 30]. A 21% increase in the proportion of vaginal deliveries in the period 2014–2016 was also observed when comparing data from 5 hospitals participating in the PPA and 8 hospitals not participating in the city of São Paulo/Brazil [12].

Serious negative outcomes, such as maternal, fetal and neonatal deaths, severe maternal morbidity and maternal and neonatal near miss, presented low frequency and with non-significant differences between the two models of care. However, women in the standard of care model presented almost twice the proportion of early term births, most of them associated with antepartum caesarean sections. It is estimated that, in Brazil, about 35% of live births are early term [31], corresponding to more than 300 thousand early term births per year, with a higher prevalence in places with caesarean rates above 80% [32]. In a previous nationwide Brazilian study, early term births, especially non-spontaneous births, were associated with several negative outcomes, such as oxygen use, neonatal ICU admission and neonatal death [31].

Women in the PPA model of care reported higher frequency of any type of violence and lower frequency of time available to make questions and to participate in the decision-making process related to childbirth. These differences were not statistically significant, but are worrisome. Recent publications have focused on the importance of involving women in the formulation and implementation of childbirth care models based on the women’ s needs, seeking a positive experience of childbirth [33, 34]. The PPA itself was implemented in response to a lawsuit brought by the women's movement in the State of São Paulo/Brazil that demanded actions to reduce unnecessary CS in private hospitals. The PPA is an ongoing project and it is probable that after the first implementation phase, this new model of care, that was shown effective in reducing caesarean sections, still needs improvements in the quality of care. A study comparing the results of the PPA in 12 hospitals in 2017 with the results of childbirth care in the private sector in the period 2011 to 2012 showed an increase in the use of recommended practices, although not all reaching a satisfactory level, and the persistent use of non-recommended practices [35].

This study has some limitations. As we used data from only one hospital, we cannot extrapolate the observed results to the set of hospitals participating in the PPA, as the local contextual characteristics may have affected the implementation of the recommended actions and the observed effects [36]. As the study excluded women who did not speak Portuguese or had hearing loss, the results are also not applicable to these women. In addition, the study is probably underpowered to detect differences in less frequent results due to the small sample size.

Costs related to the implementation and maintenance of the PPA, such as structural reforms, acquisition of equipment, training of staff, and hiring a multidisciplinary team, were not included, as they were not available. Hospital managers, health insurance companies and the project coordination should consider these expenses when deciding to implement the PPA in new maternity services.

We restricted our analysis to women from Robson's group 1 to 4, the current target group of PPA in all hospitals participating in the project [11]. Robson's groups 1 to 4 represent the majority of Brazilian women [30], but excludes all multiple, premature pregnancies, with non-cephalic presentations and, most important, women in group 5 (multiparous women with previous caesarean section and single, full-term, cephalic gestation). Women in group 5 represent more than a quarter of women assisted in private hospitals and one of the groups that contributed most to the rate of caesarean section in Brazilian hospitals [30]. Women with a previous caesarean section are an increasingly important determinant of the overall CS rate in low and middle-income countries [21]. In developed countries, the increasing use of repeat caesarean sections is the factor that has most contributed to the high rates of caesarean sections in these countries [37]. Therefore, the exclusion of women with a previous CS from the target population of the PPA is an important limitation in the efforts to reduce the global rate of CS in Brazilian private hospitals.

A previous study that used an analytical model to evaluate the cost-effectiveness of vaginal delivery in primiparous and low-risk multiparous women in Brazil found greater cost-effectiveness of vaginal birth in primiparous women and greater cost-effectiveness of caesarean sections in multiparous women with previous caesarean [38]. However, the prevalence of most of the outcomes used in the study economic model were obtained in international studies, and therefore, the cost-effectiveness of interventions that promote vaginal births after a caesarean section in Brazilian services is still a gap to be explored in future studies.

Finally, we used a short time horizon. There is evidence of negative medium and long-term effects associated with caesarean section, both for women and newborns [3, 4]. There is also evidence of more neonatal complications after hospital discharge in early term births, more frequently observed in the standard of care model.

## Conclusion

The PPA model of care was cost-effective in reducing caesarean sections in women in Robson's group 1 to 4 assisted in a Brazilian private hospital, with a higher occurrence of women in labor, with induction of labor, and a lower proportion of early term births.

In this hospital, the cost of uncomplicated vaginal births and caesarean sections was the parameter with the greatest impact on the cost-effectiveness ratio of the PPA model of care. Greater reductions in the rate of caesarean sections and neonatal complications, especially in vaginal births, would also lead to more favorable economic results.

New studies in Brazilian hospitals located in other macro-regions, in private hospitals not belonging to health insurance companies, and with the inclusion of women with previous caesarean section, are necessary to expand knowledge on the subject and to base future strategies for reducing caesarean sections in public and private hospitals in the country.

## Data Availability

All data generated or analyzed during this study are included in this published article.

## Abbreviations

ANS: National Supplementary Health Agency
BMI: Body Mass Index
ICU: Intensive Care Unit
IHI: Institute for Health Improvement
MNM: Maternal Near Miss
NICU: Neonatal Intensive Care Unit
NNM: Neonatal Near Miss
PPA: Adequate Childbirth Project
SMM: Severe Maternal Morbidity
WHO: World Health Organization
